# TMPRSS2 Isoform 1 Activates Respiratory Viruses and Is Expressed in Viral Target Cells

**DOI:** 10.1371/journal.pone.0138380

**Published:** 2015-09-17

**Authors:** Pawel Zmora, Anna-Sophie Moldenhauer, Heike Hofmann-Winkler, Stefan Pöhlmann

**Affiliations:** Infection Biology Unit, German Primate Center, Göttingen, Germany; NIH, UNITED STATES

## Abstract

The cellular protease TMPRSS2 cleaves and activates the influenza virus hemagglutinin (HA) and TMPRSS2 expression is essential for viral spread and pathogenesis in mice. Moreover, severe acute respiratory syndrome coronavirus (SARS-CoV) and other respiratory viruses are activated by TMPRSS2. However, previous studies on viral activation by TMPRSS2 focused on a 492 amino acids comprising form of the protein (isoform 2) while other TMPRSS2 isoforms, generated upon alternative splicing of the tmprss2 mRNA, have not been characterized. Here, we show that the mRNA encoding a TMPRSS2 isoform with an extended N-terminal cytoplasmic domain (isoform 1) is expressed in lung-derived cell lines and tissues. Moreover, we demonstrate that TMPRSS2 isoform 1 colocalizes with HA and cleaves and activates HA. Finally, we show that isoform 1 activates the SARS-CoV spike protein for cathepsin L-independent entry into target cells. Our results indicate that TMPRSS2 isoform 1 is expressed in viral target cells and might contribute to viral activation in the host.

## Introduction

Respiratory viruses pose a significant threat to human health. In particular, annual influenza epidemics are associated with several hundred thousand deaths every year, and interspersed pandemics may wreck even greater havoc [1], as documented by the 1918 Spanish influenza, which caused 30 to 50 million deaths [2]. Antiviral drugs against influenza are available but their effectiveness is compromised by frequent acquisition of viral resistance. Moreover, no drugs with broad antiviral activity are available to combat emerging and highly virulent respiratory viruses, including severe acute respiratory syndrome coronavirus (SARS-CoV) and Middle East respiratory syndrome (MERS) CoV. In order to close this gap, novel antiviral strategies are being sought, which allow inhibition of a broad spectrum of viruses and which are associated with a high barrier against resistance development. Host cell factors which are essential for viral spread but dispensable for cellular survival are attractive targets for such approaches to antiviral therapy.

The surface proteins of influenza A viruses (FLUAV) and coronaviruses, termed hemagglutinin (HA) and spike (S), respectively, facilitate viral binding to host cells and fusion of the viral envelope with a host cell membrane [3]. These proteins are synthesized as inactive precursors and are converted by host cell proteases into their active forms [4], a process referred to as activation in the remainder of the manuscript. Activation is essential for viral infectivity and the responsible enzymes are potential targets for antiviral therapy. Recent work indicated that several respiratory viruses hijack the type II transmembrane serine protease (TTSP) TMPRSS2 for their activation. Thus, TMPRSS2 was shown to cleave and activate the HA proteins of diverse FLUAV in culture [5–8] and studies with tmprss2-deficient mice indicated that TMPRSS2 expression is essential for spread and pathogenesis of FLUAV [9–11]. Moreover, augmented TMPRSS2 expression was found to be associated with increased risk of severe influenza upon infection with the 2009 H1N1 pandemic virus and with increased susceptibility to H7N9 FLUAV infection [12]. Finally, TMPRSS2 was shown to activate diverse CoVs [13–16], parainfluenza virus [17], human metapneumovirus [18] and hepatitis C virus [19] in cell culture and might contribute to viral spread in the host. Remarkably, the absence of TMPRSS2 does not compromise development or homeostasis [20], indicating that TMPRSS2-specific inhibitors might exert broad antiviral activity without causing substantial unwanted side effects.

Alternative splicing of the messenger RNAs produced from several TTSP genes has been reported [21,22], and may result in the production of isoforms with different functional properties. For instance, the presence of an alternative first exon in the corin mRNA can alter surface localization of the protein and conversion of the zymogen form into the mature enzyme [21]. It has been suggested that the tmprss2 transcript can be alternatively spliced [23,24], which may result in the production of two isoforms which differ only in the N-terminal, cytoplasmic tail: Isoform 1 contains 37 amino acids in its tail which are not present in isoform 2. However, previous analyses of TMPRSS2 in the context of viral infections have exclusively focused on isoform 2 while expression of isoform 1and its ability to cleave and activate the surface proteins of respiratory viruses has not been assessed.

Here, we show that mRNA encoding isoform 1 is expressed in certain lung-derived cell lines and tissues and that the protein can activate FLUAV and the S proteins of SARS-CoV and MERS-CoV (SARS-S, MERS-S), suggesting that isoform 1 could promote viral spread in the infected host.

## Materials and Methods

### Bioinformatic analysis

Nucleotide and amino acid sequences were analyzed using the BLAST server at NCBI. The alignment of the amino acid sequences of the N-termini of TMPRSS2 isoform 1 (NP_001128571.1) and isoform 2 (NP_005647.3) was constructed using the Clustal Omega software.

### Plasmid construction

Expression plasmids for H1 and H3 FLUAV HA, SARS-S, MERS-S, ACE2 and DESC1 have been described previously [25]. For construction of the plasmids containing TMPRSS3 and TMPRSS2 isoform 2, the indicated genes were PCR-amplified from previously described plasmids and cloned into pCAGGS via the EcoRI and XhoI restriction sites. To PCR-amplify TMPRSS2 transcript variant 1, an oligonucleotide (Sigma Aldrich) encoding the extended N-terminal sequences, 5’-ATGCCCCCTGCCCCGCCCGGAGGTGAAAGCGGGTGTGAGGAGCGCGGCGCGGCAGGTCATATTGAACATTCCAGATACCTATCATTACTCGATGCTGTTGATAAAGCAAG-3’ was employed as 5’ primer and TMPRSS2 transcript variant 2 was used as template. Subsequently, the PCR-amplificate was cloned into pCAGGS via the EcoRI and XhoI restriction sites. Expression plasmids for proteases with an N-terminal myc-tag were generated by PCR using the above-described plasmids as templates, as reported previously [25]. The identity of all PCR-amplified sequences was verified by automated sequence analysis.

### Cells and viruses

The cell lines 293T (ATCC CRL-3216), Calu-3 (ATCC HTB-55), Caco-2 (ATCC HTB-37), EA-hy (ATCC CRL-2922), BEAS-2b (ATCC CRL-9609), NCI-H292 (ATCC CRL-184), NCI-H727 (ATCC CRL-5815), A549 (ATCC CCL-185) and COS-7 (ATCC CRL-1651) were grown in Dulbecco’s modified Eagle’s medium (DMEM, PAN Biotech), while LNCaP (ATCC CRL-1740) and NCI-H358 cells (ATCC CRL-5807) were propagated in RPMI-1640 medium (PAN Biotech), and MDCK cells (ATCC CRL-2936) were grown in minimum essential medium (Gibco). All media were supplemented with 10% fetal bovine serum (Biochrome), 100 U/ml penicillin and streptomycin (PAN Biotech). The cells were cultured in humidified atmosphere containing 5% CO_2_. All cell lines were obtained from collaborators and were regularly checked for mycoplasma contamination. The FLUAV A/PR/8/34 (H1N1) and A/Panama/2007/99 (H3N2) were propagated in the chorioallantoic cavities of 10-days-old embryonated hen eggs (Valo BioMedia, Germany) for 48 h at 37°C. Thereafter, the eggs were euthanized by an overnight incubation at 4°C and the allantoic fluid was harvested. Before propagation in eggs, the FLUAV A/Panama/2007/99 was recovered from a reverse genetics system [26]. The amount of infectious units within the chorioallantoic fluid was determined by focus formation assay, as described previously [25]. In brief, serial 10-fold dilutions of samples were prepared and added onto MDCK cells. After 1 h of incubation, the medium was replaced by infection medium containing Avicel overlay and 2.5 μg/ml N-acetylated trypsin (Sigma). After an incubation period of 24 h, the cells were fixed with 4% formalin in PBS and incubated for 1 h with anti-FLUAV polyclonal antibody from goat (Millipore). Subsequently, the cells were washed and incubated for 1 h with anti-goat-HRP antibodies (KPL), washed again and incubated for 10 min with True Blue substrate (KPL). Finally, foci were counted and viral titers were calculated as focus forming units (FFU) per ml of culture supernatant.

### Analysis of mRNA expression in tissues and cells

Total RNA was isolated from human cell lines with RNeasy Mini Kit (Qiagen), as recommended by the manufacturer. The Human MTC™ Panel I (Clonetech) cDNA was used to analyze mRNA expression in human organs. This cDNA was obtained from pooled tissue samples from 1–15 Caucasian donors aged 18–69 and representing both sexes. Using a Cloned AMV First-Strand cDNA synthesis kit (Invitrogen) and random hexamers, the first strand cDNA synthesis was performed from 1 μg of total RNA, previously treated with DNaseI (Roche), according to the manufacturer’s protocol. The subsequent PCR was performed with Taq polymerase (New England Biolabs) using gene-specific primers for tmprss2 transcript variant 1 (forward 5’ GTG AAA GCG GGT GTG AGG A 3’ and reverse 5’CTG TGC GGG ATA GGG GTT TT 3’), tmprss2 transcript variant 2 (forward 5’GCG AGG GGC GGG GAG CGC C 3’ and reverse 5’ GGT AGT ACT GAG CCG GAT GC 3’) and GAPDH (forward 5’ ATG GGG AAG GTG AAG GTC GG 3’ and reverse 5’ ATA CTT CTC ATG GTT CAC AC 3’). All PCRs were run for 40 cycles of 30 sec denaturation at 95°C, 30 sec annealing at 58°C, and 30 sec elongation at 72°C. Amplicons were analyzed by agarose gel electrophoresis.

### Western blot analysis

For analysis of the expression of the TMPRSS2 isoforms, 293T cells were seeded into 6-well plates at a density of 2.8 x10^5^ cells/well, cultivated for 24 h and then transfected with plasmids encoding the proteases equipped with an N-terminal myc tag or transfected with empty plasmid as control. After overnight incubation, the medium was replaced with fresh DMEM, and at 48 h post transfection the cells were washed with phosphate-buffered saline (PBS), resuspended in 100 μl of 2 x sodium dodecyl sulphate (SDS) loading buffer per well and then heated at 95°C for 30 min. Protein samples were separated by SDS-PAGE and blotted onto a nitrocellulose membrane (Hartenstein). TMPRSS2 isoforms were detected using a mouse anti-myc antibody (Biomol) as the primary antibody and a horseradish peroxidase (HRP)-coupled antibody (Dianova) as the secondary antibody. Expression of β-actin, detected with anti-β-actin antibody (Sigma Aldrich), served as a loading control. Bound antibodies were detected using ECL Prime Western blotting detection kit (Amersham), according to the manufacturer’s instructions. Image acquisition was performed with a ChemoCam Imager (Intas). For analysis of TMPRSS2 isoform expression by flow cytometry, 293T cells were transfected with plasmids encoding TMPRSS2 isoforms, as described above. At 48 h post transfection, the cells were detached, incubated with ice-cold ethanol for 10 min and stained with anti-TMPRSS2 antibody (Santa Cruz Biotechnology) diluted in 1% saponin for 30 min. The mouse IgG1 (R&D Systems) served as isotype-matched control. Thereafter, the cells were washed three times with PBS and incubated with an AlexaFluor647-coupled anti-mouse antibody (Dianova) diluted in 1% saponin. After 30 min of incubation with secondary antibody, the cells were washed two times with PBS and then fixed with 2% paraformaldehyde. The staining was analyzed with an LSRII flow cytometer (BD Biosciences) and FCS Express 4 Flow Research Edition software (DeNovo Software).

### Analysis of SARS-S and FLUAV HA cleavage by TMPRSS2 isoforms

To determine cleavage of viral glycoproteins mediated by TMPRSS2 isoforms, 293T cells were seeded in 6-well plates at 2.8 x10^5^ cells/well, cultured for 24 h and then cotransfected with 6 μg of plasmid encoding FLUAV HA of the H1 or H3 subtype or SARS-S with a C-terminal V5 tag and 0.1 μg of plasmid encoding the indicated proteases, employing the calcium phosphate transfection protocol. At 48 h post transfection, the cells were harvested in PBS and treated with PBS or 250 μg/ml tosylsulfonyl phenylalanyl chloromethyl ketone (TPCK) trypsin (Sigma Aldrich) for 10 min at 37°C and processed for Western blot analysis as described above. The SARS-S protein with a C-terminal V5 tag was detected by staining with mouse monoclonal antibody reactive against the V5 tag (Invitrogen), followed by incubation with an HRP-coupled anti-mouse secondary antibody (Dianova). The FLUAV HA cleavage was detected by staining with a goat anti-FLUAV polyclonal antibody (Millipore) for H1 subtype or with a rabbit anti-H3 HA serum (Immune Technology) and HRP-coupled anti-goat or anti-rabbit antibodies (Dianova), respectively. As a loading control, the expression of β-actin was detected with anti-β-actin antibodies (Sigma Aldrich).

### Activation of SARS-S- and MERS-S-mediated virus-cell fusion by TMPRSS2 isoforms

For the analysis of the activation of SARS-S and MERS-S for virus-cell fusion, we employed a previously described retroviral pseudotyping system [25,27]. Briefly, 293T cells were seeded in T25 cell culture flasks at a density of 7.0 x10^5^ cells/flask, cultured for 24 h and then transfected by calcium phosphate precipitation with plasmids encoding for MLV gag-pol (3 μg), a firefly-luciferase harboring MLV vector (6 μg), and a plasmid encoding for SARS-S (3 μg). The transfection medium was replaced by fresh DMEM after overnight incubation, and vector-containing supernatants were harvested at 48 h post transfection by filtration through 0.45 μm filters, aliquoted and stored at -80°C. For analysis of SARS-S-/MERS-S-mediated entry, 293T cells were transiently cotransfected with plasmids encoding ACE2 or DPP4 (2 μg), respectively, and the indicated proteases (0.3 μg) or an empty plasmid as a control. The transfection medium was changed after overnight incubation, the cells were resuspended in fresh DMEM and seeded into 96-well plates. At 48 h post transfection, the cells were preincubated with dimethyl sulfoxide (DMSO) or 10 μM cathepsin B and L inhibitor MDL 28170 (Calbiochem) for 1 h and then incubated with 50 μl of infectivity-normalized pseudotypes for 8 h. Thereafter, 50 μl of fresh medium was added and the luciferase activities in cell lysate were determined at 72 h post transduction using a commercially available kit (Beetle Juice (PJK)).

### Proteolytic activation of influenza A viruses mediated by TMPRSS2 isoforms

To determine whether the TMPRSS2 isoforms can activate FLUAV, 293T cells were seeded in 6-well plates at a density of 2.8 x10^5^ cells/well and cultured for 24 h. Then, the cells were transiently transfected with 6 μg of plasmids encoding the proteases or empty plasmid, which served as a control, using the calcium phosphate transfection method. After overnight incubation, the medium was replaced by fresh DMEM. At 24 h post transfection, the cells were gently washed with PBS and then incubated with DPBS with Mg^2+^ and Ca^2+^ (PAN Biotech) supplemented with 0.2% bovine serum albumine (BSA) (MACS Miltenyi Biotec) containing FLUAV A/PR/8/34 or A/Panama/2007/99 at a multiplicity of infection (MOI) of 0.01 or 0.1, respectively. After 1 h of incubation at 37°C in a humidified atmosphere, the cells were gently washed with PBS, and fresh infection medium (DMEM supplemented with 0.2% BSA, penicillin and streptomycin) was added. To analyze virus release, the culture supernatants were collected at 48 h post infection. The amount of infectious units within the culture supernatants was determined by focus formation assay, as described above.

### Cellular localization of TMPRSS2 isoforms

To analyze the cellular localization of TMPRSS2 isoforms, COS-7 cells were transiently transfected with 6 μg of plasmids encoding TMPRSS2 isoforms or empty plasmid as control. After overnight incubation, the transfection medium was replaced with fresh DMEM. At 24 h post transfection, the cells were washed with PBS, and incubated for 1 h with DPBS with Ca^2+^ and Mg^2+^, supplemented with 0.2% BSA, containing FLUAV at an MOI of 0.5. Thereafter, the cells were washed with PBS and fresh infection medium was added. At 24 h post infection, the cells were fixed with ice cold methanol, blocked with 3% BSA for 1 h, and then stained with mouse anti-TMPRSS2 and rabbit-anti-PR8 HA antibodies (Santa Cruz and Sino Biological, respectively). After 1 h of incubation with primary antibodies, the cells were washed three times with PBS, and incubated for 1 h with anti-mouse and anti-rabbit secondary antibodies, coupled to Rhodamine Red-X and FITC (Dianova), respectively. After three final washing steps, the cells were stained with Vectashield mounting medium (Vector Laboratories) and analyzed with a Zeiss LSM 5 laser scanning microscope. Image capture was performed with Pascal Software (Zeiss) and Fiji software was used for image analysis and calculation of Pearson Correlation Coefficient.

## Results

### Expression and autocatalytic activation of TMPRSS2 isoforms in transfected cells

**Fig 1:**
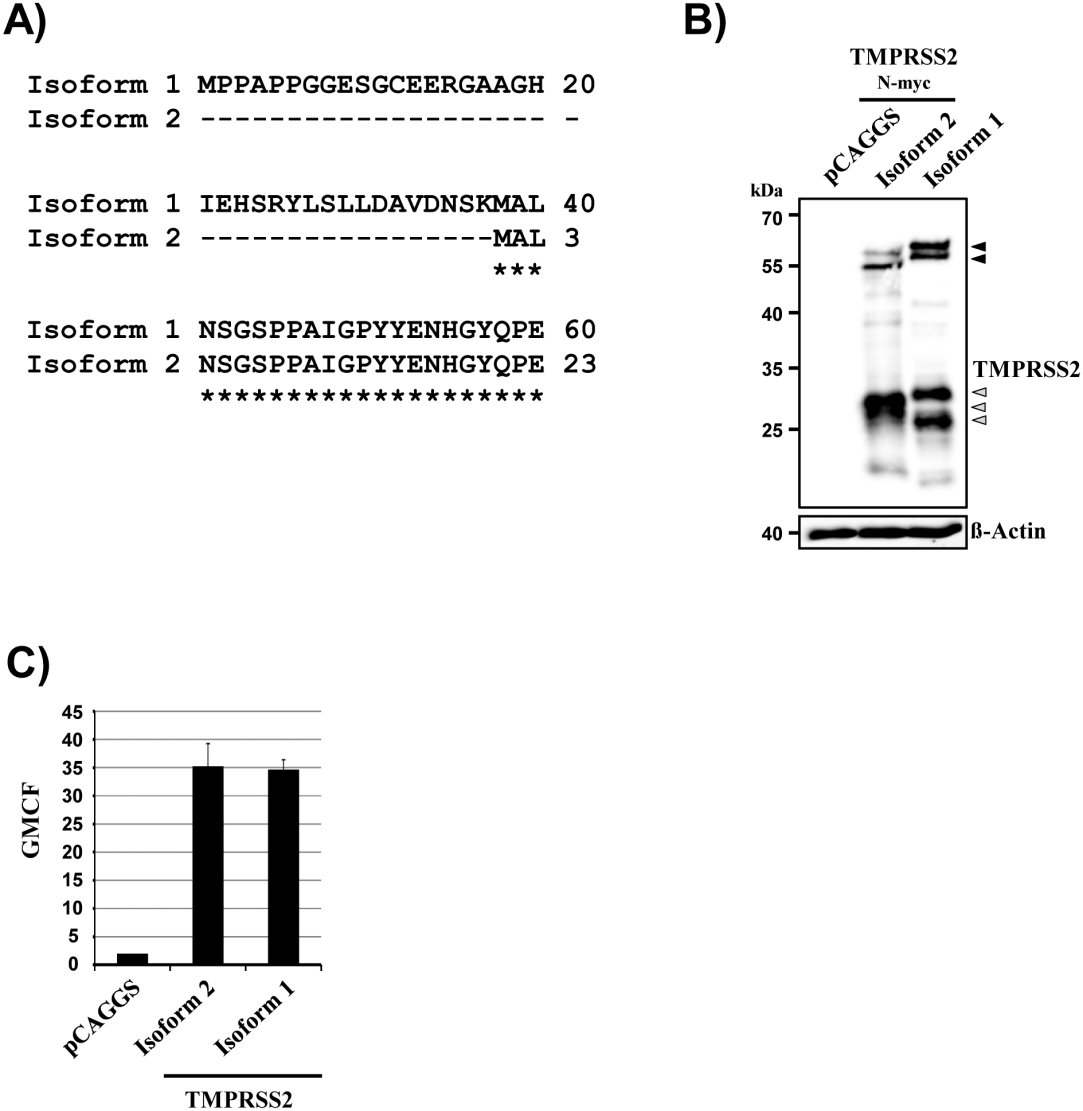
Expression of TMPRSS2 isoforms 1 and 2. (A) Sequence alignment of the N-termini of TMPRSS2 isoforms 1 and 2. Identical amino acids are marked with stars. Amino acids absent in isoform 2 are marked with ‘–‘. (B) Plasmids encoding TMPRSS2 isoform 1 and isoform 2, both equipped with an N-terminal myc tag, were transiently transfected into 293T cells. Empty plasmid (pCAGGS) served as a negative control. Protease expression in cell lysates was detected via Western blotting with anti-myc antibody. The β-actin expression served as a loading control. Black-filled arrowheads indicate the zymogen forms, while grey-filled arrowheads highlight cleavage products resulting from protease activation. (C) 293T cells were transfected as described for panel B but protease expression was determined using flow cytometry with an anti-TMPRSS2 antibody. The geometric mean channel fluorescence (GMCF) measured in a representative experiment performed with triplicate samples is shown. Error bars indicate standard deviations. Similar results were obtained in two independent experiments.

Alternative splicing of the tmprss2 transcript is believed to generate mRNAs encoding for at least two different isoforms of the protein, isoforms 1 and 2 [23,24]: Both isoforms contain identical transmembrane and extracellular domains but isoform 1 exhibits an extended N-terminal cytoplasmic domain, harboring a 37 amino acids comprising sequence which is not present in isoform 2 (Fig 1A). Isoform 2 has been shown to activate the envelope proteins of several viruses while it is at present unknown whether isoform 1 can also act as a viral activator. Moreover, it is unknown whether the isoforms are differentially expressed in viral target cells. To address these questions, we cloned the transcript variants encoding for each individual isoform and analyzed whether the proteins are efficiently expressed in transfected cells. The expression levels of TMPRSS2 isoforms 1 and 2 were comparable as determined by Western blot (Fig 1B) and flow cytometry (Fig 1C) and unprocessed isoform 1 exhibited a slightly higher molecular weight than its counterpart (Fig 1B), in keeping with expectations (expected molecular weight isoform 1, 57.7 kDa; expected molecular weight isoform 2, 53.9 kDa). Both proteins were autocatalytically activated (i.e. the inactive zymogen form was cleaved between the C-terminal protease domain and the remainder of the protein, which allows the protease domain to undergo conformational changes and to transit into an active state), as evidenced by the presence of N-terminal cleavage products. A single N-terminal fragment was observed for isoform 2 while two fragments were detected for isoform 1 (Fig 1B), suggesting that the presence of an extended N-terminus in isoform 1 does not alter the efficiency of TMPRSS2 auto-activation but might change cleavage specificity.

### Transcript variant 1 is expressed in influenza A virus target cell lines and organs

We next determined whether transcript variant 1 (which encodes isoform 1) is expressed in tissues and cell lines. For this, we employed RT-PCR with primer sets specific for tmprss2 transcript variant 1 and 2, respectively. Primers specific for variant 1 amplified their target sequence from a plasmid encoding isoform 1 but not isoform 2, confirming that the PCR was specific. Similarly, the PCR designed to amplify transcript variant 2 was negative when a plasmid encoding isoform 1 was used as template but generated the expected amplificate from cDNA prepared from several cell lines and tissues, indicating that also this PCR was specific. In this context, it should be noted that the forward primer used for detection of transcript variant 2 binds to a 5’-untranslated region which is absent from the plasmid encoding isoform 2. Therefore, the respective PCR was negative.

**Fig 2:**
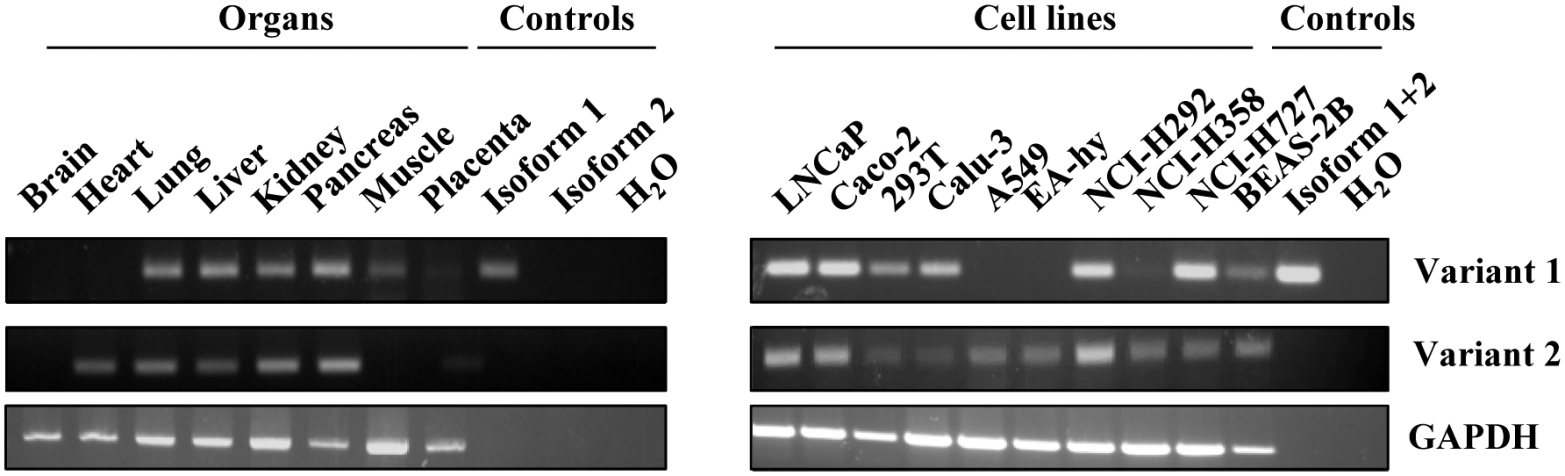
Expression of tmprss2 transcript variants in human organs and cell lines. RT-PCR analysis of the expression of tmprss2 transcript variants 1 and 2 in organ samples from adult men (left panel) or from cell lines of human origin (right panel). Expression of GAPDH was assessed in parallel. Plasmids encoding isoform 1 and 2 (left panel) or a mix of both plasmids (right panel) served as positive control. Similar results were obtained in two separate experiments.

The investigation of tissue samples revealed robust expression of transcript variant 1 in lung, kidney, liver and pancreas, which were reported to express tmprss2 mRNA (isoforms were not discriminated in previous studies) [23,28] (Fig 2, left panel). In contrast, mRNA levels in muscle and placenta were reduced and no expression of transcript variant 1 was detected in heart and brain. A similar result was obtained when expression in cell lines was determined (Fig 2, right panel): Transcript variant 1 was expressed in several but not all cell lines tested, including the lung-derived cell line Calu-3 and the colon-derived cell line Caco-2, which were previously shown to produce TMPRSS2 protein [5,6]. The expression pattern of transcript variant 2 was similar but not identical to that of variant 1. Thus, all organs and cell lines positive for transcript variant 1 also expressed variant 2 except for muscle tissue, which was negative for variant 2. Conversely, heart tissue and the cell lines A549 and EA-hy were negative for transcript variant 1 but expressed variant 2. Collectively, tmprss2 transcript variant 1 and variant 2 exhibit an overlapping but not identical expression pattern. Moreover, our results indicate that both isoforms are expressed in lung tissue and several lung-derived cell lines (including Calu-3 and NCIH292) and are thus present in FLUAV targets.

### TMPRSS2 isoform 1 and 2 colocalize with the influenza A virus hemagglutinin

**Fig 3:**
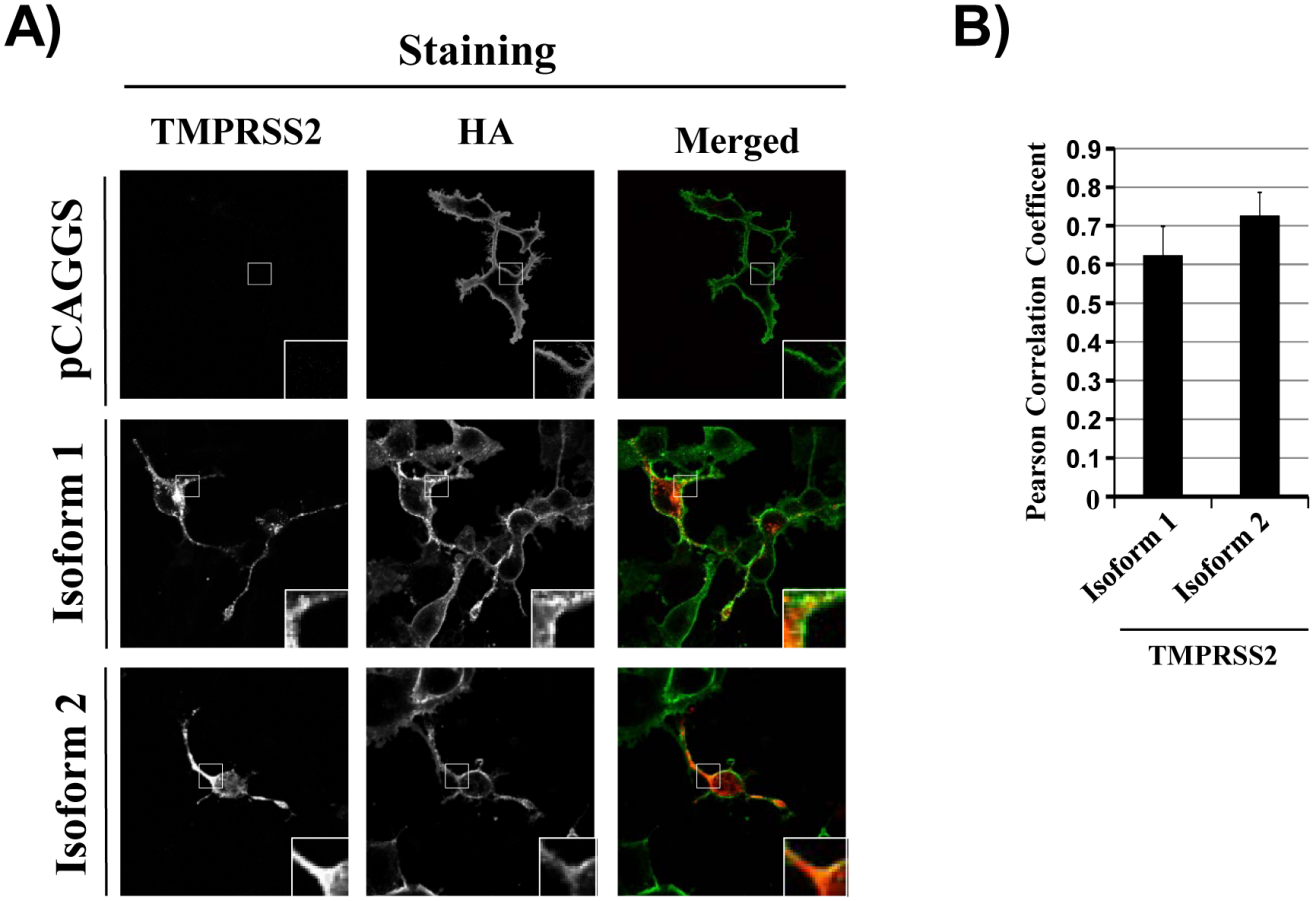
TMPRSS2 isoforms 1 and 2 colocalize with hemagglutinin. (A) COS-7 cells were transfected with plasmids encoding TMPRSS2 isoform 1 or isoform 2 or with empty plasmid which served as negative control. Subsequently, the cells were infected with FLUAV A/PR/8/34 (H1N1) at an MOI 0.5. At 24 h post infection, the cells were stained for FLUAV-HA (green) and TMPRSS2 isoforms (red) and images were taken at 63 x magnification. White squares show examples of colocalization of HA and TMPRSS2 (yellow signals) and were digitally magnified 2.5x from the original images. Similar results were obtained in three separate experiments. (B) Images obtained in (A) were analyzed with Fiji software, which allows calculation of the Pearson Correlation Coefficient (PCC), a measure for colocalization. The average PCC measured in three separate experiments is shown. For each experiment, 6–8 cells were analyzed. Error bars indicate standard error of the mean (SEM).

Activation of FLUAV by TMPRSS2 in infected cells requires that HA comes into contact with the protease. In order to address whether HA and TMPRSS2 isoform 1 colocalize, immunofluorescence staining and Fiji analysis of COS-7 cells transiently expressing TMPRSS2 isoforms and infected with FLUAV was employed. We observed colocalization of HA with both TMPRSS2 isoforms in infected cells (Fig 3A and 3B). However, we also noted differences in the cellular localization of isoform 1 and 2: although both isoforms were found at or near the cell surface, where they colocalized with FLUAV HA, more cytoplasmic puncta were observed in cells expressing isoform 1 as compared to isoform 2, which was more homogenously distributed within the cells (Fig 3A). Thus, the presence of an extended N-terminus in isoform 1 does not interfere with HA colocalization but might modestly alter the intracellular localization of TMPRSS2.

### TMPRSS2 isoform 1 cleaves and activates the glycoproteins of influenza A viruses

**Fig 4:**
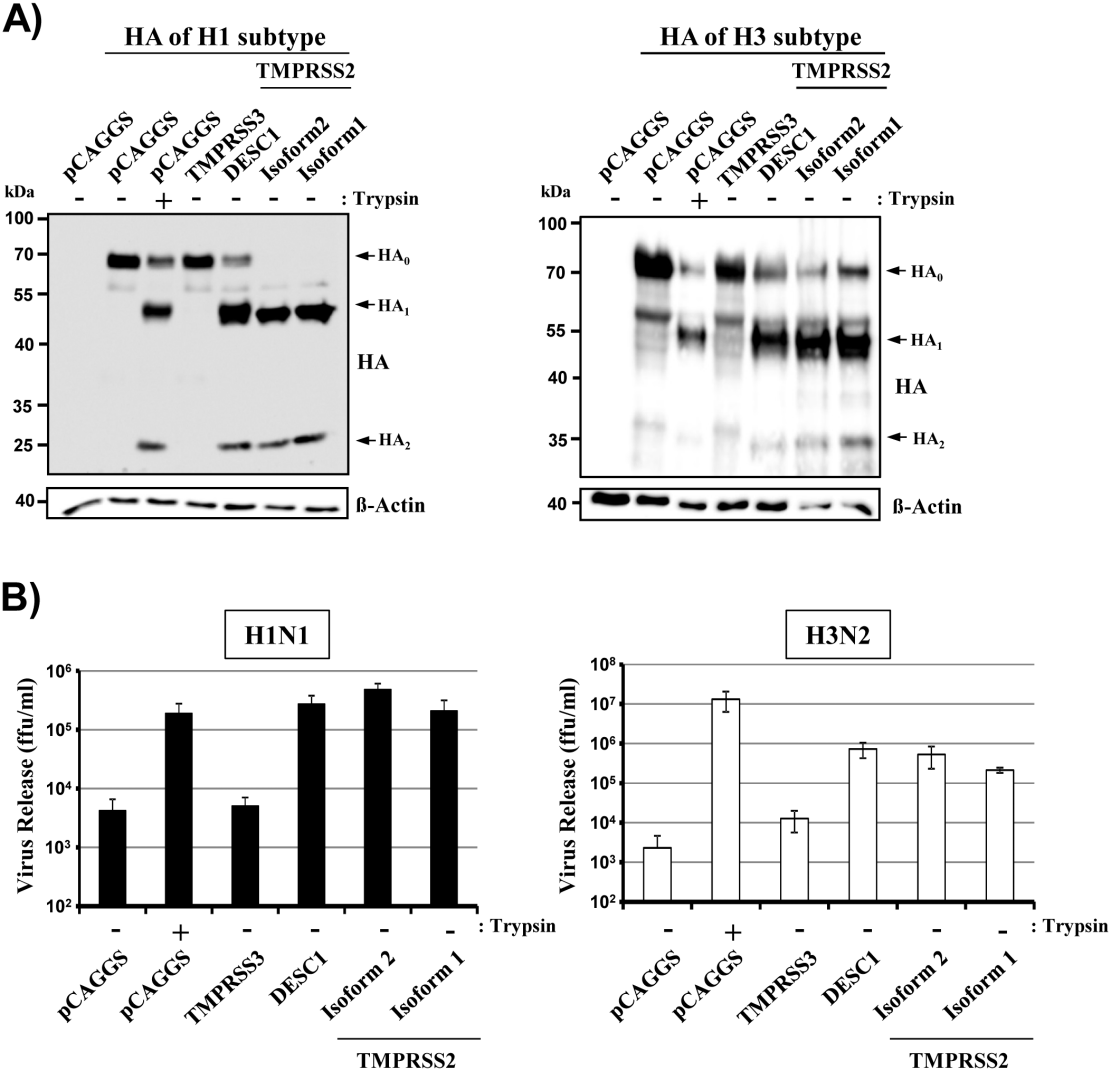
TMPRSS2 isoform 1 cleaves and activates the influenza virus hemagglutinin. (A) Expression plasmids encoding FLUAV HA subtypes H1 (left) and H3 (right) and the indicated proteases or empty plasmid (pCAGGS) were transiently cotransfected into 293T cells. At 48 h post transfection the cells were treated with PBS or trypsin, and HA cleavage was determined by Western blotting. Similar results were obtained in three independent experiments. The HA_0_ precursor (upper arrow) and the HA_1_ (middle arrow) and HA_2_ (lower arrow) subunits are indicated. (B) The indicated proteases were transiently expressed in 293T cells and the cells infected with FLUAV A/PR/8/34 (H1N1) at an MOI 0.01 (left) or FLUAV A/Panama/2007/99 (H3N2) at an MOI of 0.1 (right) and treated with either trypsin or PBS. At 48 h post infection, the virus release was measured by determination of infectious particles (ffu/ml) in the culture supernatant. The results of representative experiments performed with triplicate samples are shown. Error bars indicate standard deviations. Similar results were obtained in three independent experiments. ffu, focus forming units.

In order to determine whether TMPRSS2 isoform 1 can activate FLUAV, we first assessed cleavage of FLUAV HA. For this purpose, we coexpressed the HA proteins of FLUAV A/South Carolina/1/1918 (H1N1) or FLUAV A/Hong Kong/1/1968 (H3N2) and proteases. Cleavage of the HA precursor, HA_0_, was observed upon treatment of cells with trypsin and upon coexpression of DESC1 and TMPRSS2 isoform 2, in keeping with previous reports [5,25] (Fig 4A). Additionally, HA proteolysis was detected upon coexpression of TMPRSS2 isoform 1. In contrast, the proteolytic cleavage products were not observed upon treatment of cells with PBS or upon coexpression of TMPRSS3 (Fig 4A), as expected [25].

We next asked whether cleavage results in HA activation. To this end, we determined the spread of FLUAV A/PR/8/34 (H1N1) and FLUAV A/Panama/2007/99 (H3N2) in 293T cells transfected to express the indicated proteases. Treatment of cells with PBS or expression of TMPRSS3 did not promote viral spread (Fig 4B), as expected [5,25]. In contrast, expression of TMPRSS2 isoform 2 and DESC1 or treatment of the cells with trypsin boosted viral spread (Fig 4B), again in keeping with published findings [5,25]. Finally, expression of TMPRSS2 isoform 1 markedly increased viral spread to levels observed in the presence of isoform 2, and spread was markedly reduced by camostat mesylate (not shown), a serine protease inhibitor known to be active against TMPRSS2. In sum, these results indicate that isoform 1 and isoform 2 can efficiently cleave and activate FLUAV HA.

### TMPRSS2 isoform 1 cleaves and activates the spike protein of SARS- and MERS-coronavirus

**Fig 5:**
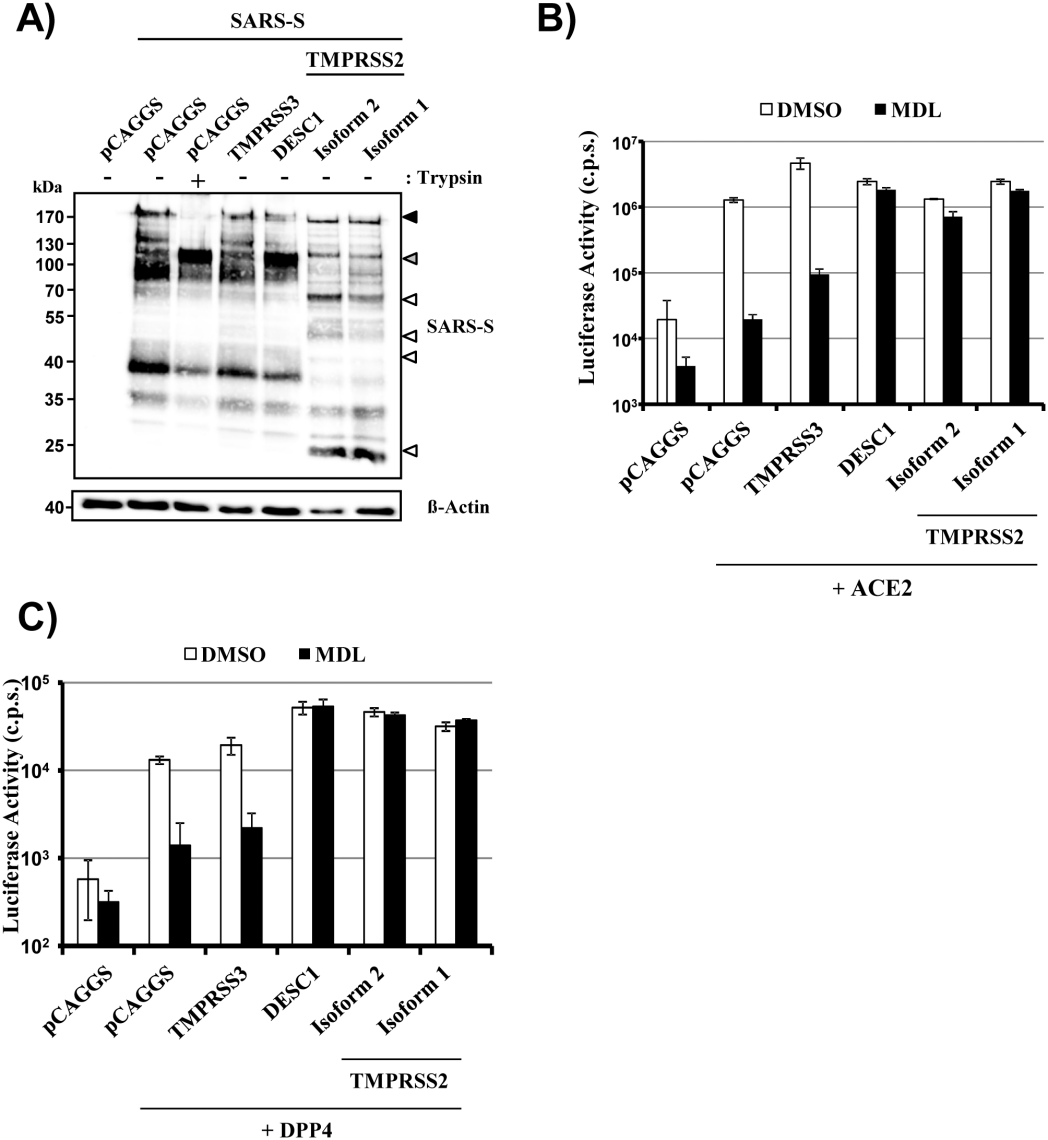
TMPRSS2 isoform 1 cleaves and activates the SARS-coronavirus spike protein. (A) 293T cells were cotransfected with plasmid encoding SARS-S with a C-terminal V5 tag and plasmids encoding the indicated proteases. At 48 h post transfection, the cells were treated with PBS or trypsin, and SARS-S cleavage was analyzed by Western blotting with a V5-specific antibody. The expression of β-actin served as a loading control. The results are representative of three independent experiments with different plasmid preparations. Black-filled arrowhead, uncleaved SARS-S; gray-filled arrowhead, S2 subunit generated by trypsin digest; white-filled arrowheads, C-terminal cleavage fragments generated by TMPRSS2. (B) To analyze SARS-S-driven virus-cell fusion, 293T cells were transiently transfected with plasmids encoding the indicated proteases and ACE2. At 24 h post transfection, the cells were pretreated with medium supplemented with DMSO or 10 μM cathepsin B/L inhibitor MDL 28170, and transduced with pseudotypes bearing SARS-S. The luciferase activities in cell lysates were analyzed at 72 h post transduction. The results of a representative experiments performed with triplicate samples are shown. Error bars indicate standard deviations. Similar results were obtained in two separate experiments. (C) MERS-S-driven virus-cell fusion was analyzed as described for panel B but 293T cells transfected to express DPP4 were used as targets. The results of a representative experiment performed with triplicate samples are shown. Error bars indicate standard deviations. Similar results were obtained in three independent experiments. c.p.s., counts per second.

Apart from FLUAV, several respiratory viruses hijack TMPRSS2 to facilitate activation of their envelope glycoprotein, including the emerging SARS-CoV [14–16] and MERS-CoV [29,30]. Activation of SARS-S and MERS-S by TMPRSS2 isoform 2 occurs during viral entry into target cells and renders this process independent from the activity of cathepsin L [14–16,29,30], an endosomal cysteine protease which can activate SARS-S and MERS-S for entry into TMPRSS2-negative cells [29–31]. We investigated whether TMPRSS2 isoform 1 can also activate SARS-S and MERS-S. Western blot analysis of protease and SARS-S or MERS-S transfected cells revealed that both TMPRSS2 isoforms processed the S proteins comparably (Fig 5A and data not shown). Moreover, SARS-S- and MERS-S-driven entry into control cells was markedly reduced by pre-incubation of cells with MDL28170, a cathepsin B and L inhibitor, and this effect was rescued by TMPRSS2 isoform 2 and DESC1 but not TMPRSS3 (Fig 5B and 5C), in keeping with published data [14–16,25,29,30]. Similarly, rescue was observed upon expression of isoform 1 (Fig 5B and 5C), indicating that the extended N-terminus is compatible with coronavirus S protein activation during entry.

## Discussion

Several host cell proteases can activate FLUAV HA in cell culture and it has thus been assumed that respiratory viruses can employ redundant proteolytic systems to ensure their activation in the host [4]. In stark contrast, recent studies indicated a central role for TMPRSS2 in viral activation: The protease was found to activate FLUAV and other respiratory viruses upon directed and upon endogenous expression in cell lines [4,6–8,14–18] and was shown to be expressed in viral target cells in the human aerodigestive tract [32]. Moreover, and more importantly, TMPRSS2 expression was required for spread and pathogenesis of FLUAV in mice [9–11]. Thus, TMPRSS2 is an important host factor for FLUAV and potentially other respiratory viruses and constitutes a potential target for antiviral intervention.

The *tmprss2* gene is located on chromosome 21 and comprises 14 exons and 13 introns [23,24]. It has been suggested that tmprss2 mRNA may be alternatively spliced [23,24] and two transcript variants were documented at NCBI, which only differ in the first exon. The encoded proteins are identical except for the presence of an extended cytoplasmic domain in isoform 1 and the impact of this extension on expression and function was unknown. The present study shows that isoform 1 is efficiently expressed in transfected cells and, in keeping with expectations, exhibits a slightly increased molecular weight relative to isoform 2. Thus, the enzymatically inactive zymogen form of isoform 1 migrated slower than its counterpart in isoform 2 (the two bands observed might be the result of differential glycosylation) and differences were also observed for the N-terminal fragment of the protease, which is generated upon autocatalytic activation. Notably, two prominent N-terminal fragments were consistently observed for isoform 1 but not for isoform 2. Thus, the extended cytoplasmic domain might give rise to the expression of different glycoforms or, maybe more likely, might alter the specificity of autocatalytic cleavage, which is essential for the enzymatic activity of TMPRSS2. In fact, an impact of the cytoplasmic domain on the autocatalytic activation has previously been reported for the TTSP corin, in which the presence of a shortened cytoplasmic tail, due to the use of an alternative exon 1, reduces plasma membrane localization and autocatalytic activation [21].

An RT-PCR designed for the amplification of a sequence specific to transcript variant 1 showed that this transcript is expressed in cell lines and tissues susceptible to FLUAV infection and previously found to be positive for tmprss2 mRNA and or protein, including lung and liver [23,28]. In general, the expression patterns of transcript variant 1 and 2 were similar but not identical, suggesting that in certain tissues only isoform 1 might be available for viral activation. Whether the mRNA expression data match the expression of protein remains to be determined and such studies are complicated by the absence of isoform-specific antibodies. No major differences in the cellular localization of the two isoforms were observed, although distribution of isoform 2 at or close to the cell surface was more homogenous than that of isoform 1, which was frequently detected within speckles. Thus, the extended cytoplasmic tail of isoform 1 slightly impacts protein localization in transfected cells and this impact might be more pronounced in cells endogenously expressing this isoform.

Our mRNA expression data suggest that at least a fraction of the TMPRSS2 molecules present in FLUAV target cells correspond to isoform 1, raising the question whether they contribute to viral activation. Our study provides an affirmative answer: Both isoform 1 and 2 colocalized with HA in transfected cells, cleaved HA upon coexpression and activated authentic FLUAV of different subtypes. Moreover, isoform 1, like isoform 2, activated SARS-S and MERS-S for entry into target cells. Thus, the presence of an extended N-terminus does not seem to alter the ability of TMPRSS2 to activate respiratory viruses, although one should take into account that transfected cells might not mirror all aspects of cells endogenously expressing isoform 1.

Collectively, our results indicate that isoform 1 of TMPRSS2 can be produced in viral target cells and likely contributes to viral activation. Therefore, approaches to suppress HA activation for influenza therapy must target both isoforms and our initial results obtained with camostat mesylate indicate that this is feasible. However, it remains to be determined whether subtle differences in cellular localization and autocatalytic activation of TMPRSS2 isoforms observed here impact sensitivity to protease inhibitors in cells endogenously expressing these isoforms.
